# The Role of Complementary and Alternative Medicine for the Management of Fibroids and Associated Symptomatology

**DOI:** 10.1007/s13669-016-0156-0

**Published:** 2016-04-25

**Authors:** Nick Dalton-Brewer

**Affiliations:** King’s Hewitt Fertility Centre, Unit 6 Business Park, King’s College Hospital NHS Foundation Trust, London, SE5 9RS UK

**Keywords:** Uterine fibroids, Infertility, Acupuncture, CAM, Cardiovascular disease, Obesity

## Abstract

This article discusses the role of complementary and alternative medicine (CAM) in the management of fibroids and associated symptomatology. Since there is such a paucity of direct research related to fibroids, conditions that are implicated in the causation of uterine fibroids and symptomatology that CAM treatments may or have been shown to make a difference are also considered.

## Introduction

Uterine fibroids are a common condition affecting women in their reproductive and post-reproductive years, with an estimated lifetime incidence of 50 % in white women and 80 % in black women.

Complementary and alternative medicine (CAM) is broadly defined as systems of medicine that fall outside of mainstream care and are external to the politically dominant health system practices [1]. In this definition, both complementary and alternative medicines are used interchangeably.

The National Centre for Complementary and Integrative Health (NCCIH) expands this definition, stating that ‘complementary medicine is used together with conventional medicine and alternative medicine is used in place of conventional medicine’ [2]. The boundaries between CAM and conventional medicine are not absolute, and specific CAM practices may, over time, become widely accepted. To confuse the matter further, certain therapies that are considered as CAM in the West are part of conventional medicine in the East. For example, acupuncture and Chinese herbal medicine are part of the conventional medical system in China. For purposes of this article, we will discuss only those therapies that are CAM as defined in the West.

Recent data shows that patient choice is integrative in that they are more likely to use both conventional medicine and CAM. Women are more likely to use CAM than men [3]. CAM use is generally greater in white and middle-class women with a higher education compared to black women or women living in areas of depravity [4].

A trial of 150,000 men and women registered with a Dutch health insurer reported that those who used integrated CAM/conventional clinic services are more likely to live longer. Interestingly, the health care cost to the integrated clinics was lower than non-integrated clinics, suggesting a role for an integrated service model in the prevention of long-term health problems [5].

## Aetiopathogenesis Related to CAM

### Diet

Dietary therapy is one of the top motivations a woman has for visiting a CAM practitioner [6].

The incidence of uterine fibroids has been shown to be greater in populations who consume more red meats such as beef and ham and alcohol. Women who drink a beer a day or more increase the risk of developing uterine fibroids by more than 50 % [7]. On the other hand, dairy consumption appears to reduce the risk of fibroids, thus suggesting a role for calcium, magnesium and phosphorus in the pathogenesis of fibroids; both calcium and butyric acid inhibit cell proliferation [8].

A diet low in fruits and vegetables has been associated with an increased risk of developing fibroids [9]. Moreover, women who consume more citrus fruits are less likely to develop fibroids, possibly due to the anti-proliferative effect of flavonoids [9].

## Isoflavones

Isoflavones are a branch of flavanoids and are mostly produced by the monophyletic family, *Fabaceae*, to which Chinese herbal medicine (CHM) such as Puerariae Lobata Radix (Gegen), and foods such as soyabeans, and other legumes belong. Isoflavone consumption differs; in the West, 2 mg per day is usual [10]. In the East, up to 50 mg per day can be consumed [11]. There is also a significant difference between herb and food isoflavone content. The main dietary source of isoflavones is soyabean; one half a cup will yield around 47 mg of isoflavones [12]. The yield from Radix Paeoniae Rubra (Chi Shao) however is about 30 times higher [12].

Isoflavones contain very small amounts of phytoestrogens that are weakly estrogenic compounds. Moreover, they are both estrogenic and anti-estrogenic depending on cell type and also have anti-oxidant properties, and perhaps, this may explain why they have not been shown to have an association or causation with uterine fibroids [13].

The flavones apigenin and luteolin can induce inhibition of uterine fibroid growth by promoting apoptosis, and quercetin, the main fruit flavonoid and anti-oxidant, also displays pro-apoptic effect on tumour cells [14, 15]. The ability of quercetin to prevent growths in human cancer cell lines with virtually no side effects to normal cells has made it an attractive candidate for further investigation [16].

Vitamin D is associated with uterine fibroids and is obtained through diet and sun exposure. Dietary sources of vitamin D include fatty fish like salmon and tuna and fortified milk. The recommended daily allowance is 20 ng/ml (600 IU) of vitamin D a day for people under 70 and over 12 months [17]. In rats, high doses of vitamin D3 shrank uterine fibroids by as much as 75 %, suggesting a role for vitamin D3. Although the doses were comparatively much larger than the recommended daily intake for humans, they were still within the limits considered safe [18].

Serum vitamin D levels have been correlated with fibroid size; deficiency correlated with the largest fibroids, whereas the highest serum vitamin D levels correlated with the smallest fibroids. Total fibroid volume correlated inversely with vitamin D in African American women. An inverse correlation was also observed in Caucasians but was not significant [19••].

Vitamin A has also been investigated, and a dose-dependent relationship was observed between vitamin A and the formation of uterine fibroids [20]. Animal sources of vitamin A appear to be primarily responsible and not sources derived from fruit or vegetables [9]. The anti-oxidant properties of vitamins C and E have not shown any association however [9].

### Stress

Stress is a threat to homeostasis. Chronic life stress is characterised by reward eating (consumption of high-energy dense and palatable foods), elevation of cortisol, and long-term weight gain correlates with the incidence of uterine fibroids [21–24].

Increases in cortisol and insulin may be a natural somatic protective response to stress, wherein the stress response both causes and is caused by a threat to homeostasis; i.e. the mechanistic trail may be convoluted. For example, stress increases activities associated with pleasure such as reward eating in order to inhibit the hypothalamic-pituitary-adrenal axis (HPA) as protective mechanism [25]. The consequent chronic suppression of cortisol levels may eventually cause insulin resistance, which in turn may result in the development of obesity, hypertension and atherosclerosis; all of which are implicated in fibroid growth [26].

### Hypertension

Evidence suggests that hypertension is involved in the pathogenesis of fibroids and precedes the development of fibroids [27]. Hypertension is significantly more likely in women with fibroids than without, and the risk of fibroid growth increases with blood pressure, in both users and non-users of hypertensive medication. For every diastolic increase of 10 mmHG, the risk of fibroid growth increases by 8 and 10 %, respectively [28, 29]. Faerstein et al. postulated that elevated blood pressure may cause smooth muscle injury and/or secretion of cytokines similarly to that found in the pathogenesis of atherosclerosis [28].

### Atherosclerosis

Atherosclerosis and uterine fibroids are both smooth muscle, monoclonal growths which may in part explain their association. High-density lipids (HDLs) which are protective of atherosclerotic changes are lower in women with fibroids than in women without, and thicker carotid intima-media have been shown to be positively associated with uterine fibroids [28, 30•].

### Obesity

The risk of developing a fibroid is between twofold and threefold in obese patients [31]. Elevated BMI and obesity correlate with patients who have both fibroids and hypertension. However, obesity does not correlate with the number of fibroids within the uterus, indicating that in addition to gonadotropins, the growth of uterine fibroids may involve other factors [32–34].

### Adipokines and Renin-Aldosterone-Angiotensin System

Both adipokines and the renin-aldosterone-angiotensin system (RAS) are expressed in the reproductive system, and increase in expression may be implicated in the pathogenesis of uterine growths and their recurrence [35–37].

The presence of uterine fibroids is associated with significantly decreased levels of the adiponectin [38]. Although adiponectin is exclusively secreted by adipose tissue, yet its expression is inversely correlated with body fat. Low adiponectin levels are implicated in the development of atherosclerosis, obesity and insulin resistance [39].

## CAM Therapies for Management of Uterine Fibroids

### Dietary Therapy

Diets related to the pathogenesis of fibroids such as red meats and high-energy dense foods should be avoided, whereas diets which prevent fibroid pathogenesis such as flavonoids, oily fish, green vegetables, citrus fruits, soya and broad beans should be promoted. These modifications not only have direct effects on fibroids but on related mechanisms. For example, hypertension is implicated in pathogenesis and reducing sodium levels can reduce blood pressure. A treatment strategy that excludes red meat, fat and animal fat, and restricts diet to fruits vegetables and poultry, and reduces sodium intake can thus reduce blood pressure and may also exert a protective effect on fibroid growth [40].

A suggested dietary therapy is outlined in Table 1. Whilst the recommendations are based on direct and indirect pathogenesis associations, they have not been subjected to large-scale trials.

**Table 1:** Dietary therapy and advice

Dietary therapy and lifestyle advice			
Inclusionary foods and drinks	Exclusionary foods and drinks	Supplements include	Lifestyle options include
Fish		Omega 3 and 6	Mindfulness meditation
Salmon		Vitamin D	Yoga
Tuna		Vitamin A	Tai Chi
Mackerel		Vitamin C	Regular exercise
Other oily fish		Vitamin E	Breathing exercises
		Iron supplements including	Dietary support
Meats			
Chicken	Beef		
Turkey	Ham		
Other white meat	Lamb		
	Other red meat		
Vegetables			
Soyabeans	Chips/crisps		
Fava beans^a^	Potatoes		
Green vegetables	Rice		
Other legumes	Sweets and chocolate		
Fruit			
Apples			
Oranges			
Tangerines			
Other citrus fruits			
Fluids			
Green tea			
Vegetable juice	Alcohol/beer		
Unsweetened fruit juice	Sweetened juice		
Milk (semi-skinned)	High-sugar drinks		

^a^Not to be consumed by people with the hereditable condition of Favism

## Herbal Medicine

The first record of CHM in gynaecology (the treatment of fertility) was written in 200 AD. However, the modern construction of a herbal formula is still based on the traditional criteria insofar as the phytokinetics and dynamics of the herbs is concerned. Integrating a medical system can prove beneficial to patients, for example, using clomiphene citrate with a traditional formula to treat anovulatory infertility arising from polycystic ovarian syndrome (PCOS) can achieve better results than using either CHM or clomiphene citrate alone [41].

### Gui Zhi Fu Ling Tang *Ramulus cinnomomi* and *Poriae cocos* decoction

The most commonly used traditional Chinese medicine (TCM) formula to treat uterine fibroids is Gui Zhi Fu Ling Tang (GFLT) [42]. This formula is effective in the treatment of dysmenorrhea either as a stand-alone treatment or in combination with progesterone receptor modulator such as mifepristone. The combination GFLT mifepristone therapy was shown to be more effective than using mifepristone alone [43•]. Three of the herbs used are known to be anti-proliferative and are involved in tumour cell apoptosis and induction of follistatin, Mu Dan Pi (Cortex Moutan), Chi Shao (Radix Paeoniae Rubra), and Tao Ren (Semen Persicae) [44–46].

#### Danshen Gegen decoction (Salviae Miltiorrhizae Radix et Rhizome and Purariae Lobatae Radix decoction)

The CHM formula Danshen Gegen decoction (DGD) has a long history of use and recently has been investigated for its anti-atherosclerotic effects. Investigative results into DGD indicate that it positively modulates key early events in atherosclerosis [47–49]. The main active components in Danshen and Gegen, tanshinones and genistein, respectively, are most likely responsible for the effects of DGD, although other factors in the formula may also be involved.

#### Genistein

The isoflavone genistein has a number of properties involved in the inhibition atherosclerosis. Genistein is an anti-inflammatory and modulates vascular inflammation [50]. Genistein also inhibits tyrosine kinases, enzymes involved in cellular growth and proliferation signal cascade; blocks platelet aggregation; and modulates genes related to cell cycle and apoptosis [51]. In addition, genistein also modulates nuclear factor-kappa B (NFkB). As a result, genistein may also be able to mediate uterine fibroid growth [52].

#### Tanshinones

The flavonoid tanshinone IIA is derived from Danshen (Salviae Miltiorrhizae Radix et Rhizome), a herb used in the treatment of hypertension, cardiovascular disease and hypertension, cancer and liver damage [53].

Tanshinone IIA attenuates atherosclerosis [54]. Its effects include downregulation of adhesion molecules, improvement of microcirculation by inducing endothelium-dependent vasodilatation in coronary arterioles through a range of neuromodulators including endothelial nitric oxide synthase (eNOS) and angiotensin II [55, 56]. Interestingly, it also inhibits vascular smooth muscle cell proliferation and decreases intimal thickening through mechanisms involving MAPK signalling pathway [57].

#### Green Tea (*Camellia sinensis*)

Twenty-five percent of green tea is comprised of the flavanols and catechins, and epigallocatechin-3-gallate (EGCG) is extracted from this group. A small randomised controlled trial (*n* = 39) found that the flavanol EGCG significantly reduced volume of uterine fibroids and improved symptoms of anaemia and blood loss [58•].

The reduction of fibroid volume is attributed in part to the inhibitory action of EGCG on catechol-*O*-methyltransferase (COMT). In comparison to surrounding myometrial tissue, COMT is elevated in uterine fibroids and involved in their pathogenesis [59]. A common genetic variation of COMT is also implicated in cardiovascular disease and high blood pressure [60, 61]. Furthermore, a reduction of atherosclerotic lesions induced by COMT in animals implies a relationship between EGCG and COMT in the treatment of this disease in humans [62].

### Acupuncture

TCM is a system of medicine with several therapies, of which TCM acupuncture is the most popular in literature. In the 1950s, electro-acupuncture was introduced to TCM. There is an abundance of evidence that acupuncture modulates a wide range of endocrinological, neurohormonal, immunological, paracrine and autocrine factors [63–65].

Acupuncture acts on a variety of therapeutic targets associated in the pathogenesis and symptomatology of fibroids. It is effective treatment for dysfunctional bleeding and chronic pelvic inflammatory disease and dysmenorrhoea [66–68] (see Table 2 for acupuncture points used in the treatment of heavy periods).

**Table 2:** Acupuncture points: heavy periods

Acupuncture points for heavy periods resulting from blood stasis		
Channel name and number	Pinyin	Actions associated with traditional use
Gall bladder 34	Yang Ling Quan	Moves blood
Ren 6	Qihai	Moves blood
Spleen 10	Xuehai	Invigorates blood
Bladder 17	Geshu	Invigorates blood
Stomach 29	Guilai	Invigorates blood in the lower abdomen
Liver 3	Taichong	Invigorates liver blood
Spleen 6	Sanyinjiao	Invigorates liver blood
Liver 8	Qu Quan	Nourishes blood
Stomach 36	Zusanli	Nourishes blood
Bladder 20	Pishu	Nourishes blood
Penetrating vessel spleen 4 + pericardium 6	Gongsun + Neiguan, respectively	Regulates blood

Maciocia [69] and Deadman et al. [70]

**Fig. 1:**
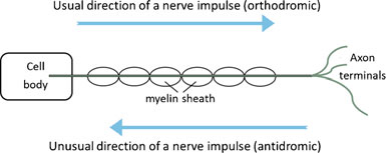
Nerve impulses (also known as an action potentials) usually travel from the cell body to the axon terminals (orthodromic). In some cases, nerve impulses travel from the axon terminals towards the cell body (antidromic)

Stener-Victorin suggested that stimulation of afferent nerve fibres may be one of the mechanisms that acupuncture exerts its effects. Such stimulation would inhibit sympathetic outflow at the spinal level. Furthermore, acupuncture also causes secretion of sensory neurotransmitters, such as substance P and calcitonin gene-related peptide (CGRP), which may effect neuronal transmission. Nerve impulses (action potentials) usually travel away from the nerve cell body, along the axon, to the axon terminals, from which the impulses are conveyed (to a subsequent neuron). This usual direction of travel is termed orthodromic. However, because acupuncture induces the secretion of sensory neuromodulators, an effect of acupuncture may induce nerve impulses to travel in the opposite direction, that is, away from the axon terminals towards the cell body. This opposite direction of travel is termed anti-dromic and may be another mechanism by which acupuncture modulates the central nervous system [71, 72] (see Fig. 1).

Whether these effects seen in mixed populations translates into improvements in bleeding and pain symptoms associated with fibroids per se remains to be seen.

Acupuncture treatments are effective in improving outcomes in overweight and obese patients. Results from animal experiments have found that acupuncture treatments reduce appetite and affect satiety at the level of the hypothalamus, thus implicating a role of energy homeostasis neurohormones such as leptin in the management of appetite by acupuncture [73]. This effect may also be a result of the pro-inflammatory cytokine downregulation involved in acupuncture treatments [74].

Both manual acupuncture and low-frequency electro-acupuncture (LFEA) can lower leptin and raise adiponectin secretion [75, 76].

#### Acupuncture and Infertility

The role of fibroids in causation of infertility is controversial. Fibroids in certain locations and sizes have been shown to lower success rates. On the other hand, irrespective of cause, infertility and its treatments result in significant stress and anxiety to patients. Women are more likely to experience guilt and hostility than fertile women, and treatment can lead to clinical anxiety and depression [77].

Fertility-related stress may also negatively affect IVF treatment [78•]. Trials have also shown that acupuncture significantly alleviates depression, anxiety and stress by modulating both specific and non-specific neurological signalling and also neuromodulators such as cortisol, prolactin, epinephrine and beta endorphin [79, 80].

The effect of acupuncture treatments for IVF/ICSI has been examined during the last decade. Whilst some trials have found significant increases in clinical pregnancy and live birth rates when acupuncture is integrated with IVF, the results of meta-analyses are inconclusive due to methodological differences and heterogeneity of populations studied [81–84]. In addition, placebo acupuncture may not be inert, which may explain some of the differences between placebo-controlled trials involving acupuncture [85].

A variety of mechanisms have been proposed to explain the anti-fertility effects of uterine fibroids. Of particular interest to CAM is an increase in myometrial contractility observed with fibroids during implantation and increase in conception rates following myomectomy in women with two or more peristaltic movements in 3 min [86].

A potential candidate marker for effectiveness of acupuncture therapy is the vasoactive modulator, CGRP, which is upregulated during the window of implantation [87]. CGRP inhibits angiotensin II and relaxes smooth muscle in the uterus [88]. Modulation of CGRP by acupuncture may inhibit uterine contractions associated with fibroids, and a trial investigating this is underway at our centre.

## Suggested Approach to the Management of Uterine Fibroids

An integrated approach using both CM and CAM is essential in providing holistic and safe and effective treatment [89, 90]. Following referral, a detailed history is taken including lifestyle, diet, family history, and signs and symptoms relating to TCM. The results of any previous investigations and treatments including CM are also reviewed. It is best practice that the diagnosis of fibroids is established using conventional medicine.

Therapeutic options depend on the outcome of consultation, investigations, interdisciplinary discussions and patient choice. CAM treatment may involve herbal, acupuncture and dietary/lifestyle choices. Insofar as CAM treatments are selected, CHM is the treatment of choice to reduce volume of uterine fibroids, and, for management of chronic conditions, whereas acupuncture is more efficient in the management of acute conditions, probably due to its modulation of neurohormones such as beta endorphins, leptin and other biochemicals. The increased frequency of acupuncture treatments potentiates the effects of acupuncture, and so, the therapy can also be used in the treatment of chronic conditions.

During the course of treatments, other problems may arise and it is important to treat what is presented by the patient at that time.

## Conclusion

A variety of CAM therapies are available, and there is an urgent need to subject them to academic rigour. A clear understanding of underlying mechanistic pathways and patient’s symptoms and needs is crucial in planning individualised therapies. An integrated CM CAM model is likely to yield better patient outcomes and reduced health care costs.
